# Genome-Wide Identification of *Sorghum bicolor* Laccases Reveals Potential Targets for Lignin Modification

**DOI:** 10.3389/fpls.2017.00714

**Published:** 2017-05-05

**Authors:** Jinhui Wang, Juanjuan Feng, Weitao Jia, Pengxiang Fan, Hexigeduleng Bao, Shizhong Li, Yinxin Li

**Affiliations:** ^1^Key Laboratory of Plant Molecular Physiology, Institute of Botany, Chinese Academy of SciencesBeijing, China; ^2^Institute of Botany, University of Chinese Academy of SciencesBeijing, China; ^3^Department of Biochemistry and Molecular Biology, Michigan State UniversityEast Lansing, MI, USA; ^4^Key Laboratory of Marine Food Quality and Hazard Controlling Technology of Zhejiang Province, College of Life Sciences, China Jiliang UniversityHangzhou, China; ^5^Beijing Engineering Research Center for Biofuels, Tsinghua UniversityBeijing, China

**Keywords:** *Sorghum bicolor*, lignin, laccase, genetic engineering, lignin modification

## Abstract

Laccase is a key enzyme in plant lignin biosynthesis as it catalyzes the final step of monolignols polymerization. Sweet sorghum [*Sorghum bicolor* (L.) Moench] is considered as an ideal feedstock for ethanol production, but lignin greatly limits the production efficiency. No comprehensive analysis on laccase has ever been conducted in *S. bicolor*, although it appears as the most promising target for engineering lignocellulosic feedstock. The aim of our work is to systematically characterize *S. bicolor* laccase gene family and to identify the lignin-specific candidates. A total of twenty-seven laccase candidates (*SbLAC1*-*SbLAC27*) were identified in *S. bicolor*. All SbLACs comprised the equivalent L1-L4 signature sequences and three typical Cu-oxidase domains, but exhibited diverse intron-exon patterns and relatively low sequence identity. They were divided into six groups by phylogenetic clustering, revealing potential distinct functions, while SbLAC5 was considered as the closest lignin-specific candidate. qRT-PCR analysis deciphered that *SbLAC* genes were expressed preferentially in roots and young internodes of sweet sorghum, and *SbLAC5* showed high expression, adding the evidence that *SbLAC5* was *bona fide* involved in lignin biosynthesis. Besides, high abundance of *SbLAC6* transcripts was detected, correlating it a potential role in lignin biosynthesis. Diverse *cis* regulatory elements were recognized in *SbLACs* promoters, indicating putative interaction with transcription factors. Seven *SbLACs* were found to be potential targets of sbi-miRNAs. Moreover, putative phosphorylation sites in SbLAC sequences were identified. Our research adds to the knowledge for lignin profile modification in sweet sorghum.

## Introduction

Sorghum [*Sorghum bicolor* (L.) Moench], as a widely adapted C4 cereal crop, is the fifth most cultivated crop around the world. It can be planted on marginal or non-arable lands owing to its favorable traits of low-input cost but high-yielding, drought-tolerance, high nutrient-, and water-use efficiency (Yuan et al., 2008). Sweet sorghum is a natural variant of common grain sorghum with greater height, higher biomass, and especially higher level of fermentable sugar in stems (Rooney et al., 2007; Calviño and Messing, 2012). It has been increasingly grown as a dedicated bioenergy feedstock offering grain, forage, sugar, and fiber simultaneously, therefore adds a new member to the family of bioenergy crops (Gill et al., 2014). In industrial production, both the starch in grain and the sugar in stem can be directly fermented for ethanol, while crop residuals are favorable lignocellulosic feedstock for ethanol production, i.e., the second generation biofuel.

The major component of lignocellulosic biomass is plant cell walls which mainly consist of cellulose, hemicellulose, and lignin. Lignin is a complex heteropolymer derived primarily from three monolignol units: *p*-coumaryl alcohol (H), coniferyl alcohol (G), and sinapyl alcohol (S) (Boerjan et al., 2003; Vanholme et al., 2010). It is usually covalently linked to cellulose and hemicellulose, conferring mechanical strength, and hydrophobicity to cell wall but increased recalcitrance to lignocellulose (Chang, 2007; Zeng et al., 2014). Several studies have demonstrated the link between reduced lignin levels and decreased recalcitrance with improved saccharification efficiency (Chen and Dixon, 2007; Jackson et al., 2008). Hence, if the lignocellulose composition of sweet sorghum can be manipulated toward lower lignin but higher cellulose, great economic, and environmental benefits will be achieved (Sticklen, 2006; Wang P. et al., 2015). And in the production practice, pretreatment of raw materials with lignolytic enzymes or biomass lignin modification has been frequently attempted in order to increase lignocellulose digestibility for higher biofuel yields. Genetic attempts always focused on down-regulating individual monolignol biosynthetic genes such as *PAL, C4H, 4CL, HCT, COMT, CCR*, and *CAD*, where altered lignin profile were achieved but accompanied by defected morphology (Boudet et al., 2003; Bonawitz and Chapple, 2010). By comparison, a little effort has been focused on laccase, which has newly certified function in catalyzing monolignols oxidation and polymerization in plant lignin synthesis (Berthet et al., 2011; Zhao et al., 2013).

Laccase (*p*-diphenol:dioxygen oxidoreductase, EC 1.10.3.2) is a member of the multicopper oxidases (MCOs) family. It has been reported to catalyze the one-electron oxidation of a wide range of substrates, coupled with the reduction of oxygen to water (Mot and Silaghi-Dumitrescu, 2012). Typical laccase contains three conserved Cu-oxidase domains, coupled with four copper ions: a mononuclear blue copper ion (Cu1) at the T1 site conferring the typical blue color, and a trinuclear copper cluster at the T2/T3 site consisting of one T2 copper ion (Cu2) and two T3 copper ions (Cu3) (Morozova et al., 2007; Giardina et al., 2010; Dwivedi et al., 2011). A total of 12 amino acid residues, including 10 histidines, and one cysteine as well as an axial methionine or leucine, have been thought to serve as copper ligands. They are housed within a set of four ungapped sequence regions L1-L4, which have been identified as signature sequences that distinguish laccase among the broader class of multicopper oxidases (Kumar et al., 2003). Laccase is widely distributed in plants, bacteria, fungi, and insects, while plant laccase is clustered in a separate clade in phylogenetic tree (Wang J. H. et al., 2015). To date, laccases have been characterized in many plants, such as Anacardiaceae, *Arabidopsis thaliana* (McCaig et al., 2005; Turlapati et al., 2011), *Brachypodium distachyon* (Wang Y. et al., 2015), *Brassica napus* (Zhang K. et al., 2013), cotton (*Gossypium arboreum*) (Wang et al., 2004), loblolly pine (*Pinus taeda*) (Bao et al., 1993), maize (*Zea mays*) (Caparrós-Ruiz et al., 2006; Liang et al., 2006b), poplar (*Populus trichocarpa*) (Ranocha et al., 1999), rice (*Oryza Sativa*) (Cho et al., 2014), ryegrass (*Lolium perenne*) (Gavnholt et al., 2002), sugarcane (*Saccharum officenarum*) (Cesarino et al., 2013), sycamore maple (*Acer pseudoplatanus*) (LaFayette et al., 1995), tobacco (*Nicotiana tabacum*) (Kiefer-Meyer et al., 1996), and yellow poplar (*Liridendron tulipifera*) (LaFayette et al., 1999). Diverse temporal and spatial expression patterns have been previously reported for plant laccases. In Arabidopsis, *LAC4* was uniquely expressed in interfascicular fibers and seed coat columella while *LAC7* in hydathodes and root hairs, *LAC8* in pollen grains and phloem, *LAC15* in seed coat cell walls, and *LAC17* in interfascicular fibers (Berthet et al., 2011; Turlapati et al., 2011). In *B. distachyon, BdLAC5* and *BdLAC6* were mainly expressed in lignified tissues (Wang Y. et al., 2015). Such different expression profile indicates tissue specific physiological/biochemical roles for laccase genes. Besides, expression of multiple laccases within one tissue has been detected as well. For example in *P. trichocarpa*, 30 *PtrLAC* transcripts were expressed in stem differentiating xylem, of which 17 are abundant, suggesting a certain level of functional redundancy (Lu et al., 2013).

Recently in 2011, plant laccase has been genetically demonstrated to participate in lignin biosynthesis, while peroxidase has always been deemed to play the major role of catalyzing monolignols oxidative polymerization (Shigeto and Tsutsumi, 2016). Experimental evidence was preliminarily derived from the Arabidopsis *LAC4* and *LAC17*, since lignin content reduced by 20 and 40% in double mutant *lac4-1lac17* and *lac4-2lac17*, respectively; On the other hand, complementation with *LAC17* restored the lignin profile of *lac17* to normal. This provided the first genetic evidence that both *LAC4* and *LAC17* contribute to the constitutive lignification of Arabidopsis stems (Berthet et al., 2011). Researches in *S. officenarum* discovered that *SofLAC* mRNA is preferentially accumulated in sclerenchymatic bundle sheaths of young internodes, and in the meanwhile, heterogenous expression of *SofLAC* in Arabidopsis was able to restore the lignin content of *lac17* mutant, demonstrating the role of *SofLAC* in lignification of sugarcane (Cesarino et al., 2013). In *B. distachyon*, the *BdLAC5*-misregulated *Bd4442* mutant line exhibited significant alterations in lignification of mature culms, with a 10% lower lignin level, a slight increase of S lignin unit frequency, and a substantial increase of measurable FA esters, indicating that *BdLAC5* is required for *B. distachyon* lignifications (Wang Y. et al., 2015). All these findings suggest that genetic manipulation of lignin biosynthesis-specific laccases is a feasible strategy for fine-tuning lignin content and/or composition.

It should be a bold and promising attempt to achieve better degradable *S. bicolor* biomass through manipulation of *S. bicolor* laccase. However, no research has ever been conducted. The objective of our work is to characterize *S. bicolor* laccases, with the long-term goal to identify *bona fide* laccases involved in monolignols oxidative polymerization. In this work, gene structure and protein domains as well as putative promoter *cis* regulatory elements were analyzed. A phylogenetic tree was constructed using the neighbor-joining method. In addition, the expression patterns of *S. bicolor* laccase genes were analyzed by quantitative RT-PCR. To sum up, twenty-seven laccase candidates were identified in *S. bicolor* genome. All laccase members have conserved copper-binding domains but are different in gene structures, indicating similar genetic origin but divergent biological functions. The potential regulation of *SbLAC* genes by TFs, miRNAs and phosphorylation was discussed. More efforts are needed to find out the *bona fide* lignin-specific laccase gene, which will shed light on modification of lignin profile in *S. bicolor*.

## Materials and methods

### Plant materials and growth conditions

In previous researches of our laboratory, comparative transcriptome combined with morpho-physiological analyses were performed to reveal the key factors responsible for differential cadmium accumulation in two contrasting sweet sorghum genotypes, among which the high-Cd accumulation one UMM EL TEIMAN (accession: PI 152873) was designated as H18 (Feng et al., unpublished data). Seeds of the sweet sorghum genotype H18 were obtained from Plant Genetic Resources Conservation Unit, the United States Department of Agriculture, Griffin, USA. The plants were grown in greenhouse with a day/night temperature regime of 25/20°C, a photoperiod of 16/8 h (light/dark), and a relative humidity of 50 ± 10%.

### Genome-wide characterization of laccase genes in *S. bicolor*

The amino acid sequences of AtLAC1 to AtLAC17, ZmLAC1 to ZmLAC5, GaLAC1, SofLAC, BnTT10-1, BdLAC5, PtLAC3, PtLAC90, and PtLAC110 (Database accession numbers were listed in Supplemental Table 1) were used as queries for local BLASTP search against the Phytozome *S. bicolor* v3.1 proteome database (https://phytozome.jgi.doe.gov/pz/portal.html#!info?alias=Org_Sbicolor). The resulted peptide sequences were verified while re-blasted in NCBI (https://blast.ncbi.nlm.nih.gov/Blast.cgi) and checked on SMART (http://smart.embl-heidelberg.de/smart/set_mode.cgi?NORMAL=1). Those possessing typical Cu-oxidase domain were predicted to be laccase candidates after exclusion of monocopper oxidase-like proteins and *L*-ascorbate oxidase homologs.

Putative signal peptide cleavage sites and subcellular locations were predicted by SignalP 4.1 server (http://www.cbs.dtu.dk/services/SignalP/) and TargetP 1.1 server (http://www.cbs.dtu.dk/services/TargetP/), respectively. Potential glycosylation sites and phosphorylation sites were separately analyzed through online NetNGlyc 1.0 Server (http://www.cbs.dtu.dk/services/NetNGlyc/), YinOYang 1.2 server (http://www.cbs.dtu.dk/services/YinOYang/), and NetPhos 2.0 Sever (http://www.cbs.dtu.dk/services/NetPhos/). Visualization of the intron-exon structure of *SbLAC* genes was conducted by GSDS 2.0 server (http://gsds.cbi.pku.edu.cn/). Multiple amino acid sequences were aligned by Genestudio software (http://www.genestudio.com/).

### Phylogenetic analysis

A neighbor-joining phylogenetic tree was constructed by MEGA (http://www.megasoftware.net/) to gain insights into the evolutionary relationships between SbLACs and other plant laccases, with bootstrap tests for 1,000 replicates. Those previously characterized laccases like AtLAC1 to 17, ZmLAC1 to 5, GaLAC1, SofLAC, BnTT10-1, BdLAC5, PtLAC3, PtLAC90, and PtLAC110 were included in the analysis.

### RNA extraction and quantitative RT-PCR

Roots and internodes at three developmental stages (internodes 2, 6, and 12 from the bottom up, corresponding to the mature, developing and young phase, respectively) as well as leaves to corresponding internodes were collected from 50-days old H18 plants and were put into liquid nitrogen immediately. Total RNA was extracted with Trizol reageant (Transgen, China) followed by RNase-free DNase I (Fermentas, Lithuania) digestion. The first strand cDNA was afterwards synthesized by *TransScript*® reverse transcriptase (Transgen, China), all according to the manufacturer's instructions. Gene-specific primers (Information is available in Supplemental Table 2) were designed for analysis of laccase expression profile, with specificity being confirmed. The qRT-PCR experiment was performed with an Mx3000P™ real-time PCR system (Agilent, USA), using THUNDERBIRD SYBR® qPCR mix (Toyobo, Japan). The relative gene expression levels were calculated by the 2^−ΔΔCt^ method (Livak and Schmittgen, 2001), while 18S rRNA was chosen as the internal control. Each sample has three independent replicates.

### *In silico* analysis of *SbLAC* promoter sequences

The promoter sequences of *S. bicolor* laccases were investigated for potential *cis*-acting regulatory elements with PlantCARE (Plant *cis*-Acting Regulatory Elements, http://bioinformatics.psb.ugent.be/webtools/plantcare/html/). The identified elements were sort out according to their reported functions.

### Prediction of sbi-miRNA target laccase genes

The transcript sequences of 27 SbLACs were uploaded to web-based psRNATarget server (http://plantgrn.noble.org/psRNATarget/?function=3) for identification of potential targets corresponding to the preloaded *S. bicolor* miRNAs (241 published miRNAs from miRBase Release 21, June 2014). Sequences with a cut-off score ≤3 were chosen as putative targets.

## Results

### Twenty seven laccase genes were identified in *S. bicolor* genome

In order to identify *S. bicolor* laccases *in silico*, blastp search was performed using those well-characterized laccases from *Arabidopsis, B. distachyon, B*. *napus, G*. *arboretum, Z*. *mays, P*. *trichocarpa, S*. *officenarum* as queries against *S. bicolor* protein database, which output 57 *S. bicolor* hits. Then they were blasted in NCBI and checked on SMART, the results of which showed that 9 of them were monocopper oxidase-like proteins, 15 were deduced as *L*-ascorbate oxidase homologs (Details in Supplemental Table 3), while the other 33 with typical Cu-oxidase domains were considered as potential laccases. Taken alternative splicing in Sobic.003G111900, Sobic.003G357500, and Sobic.003G357700 into consideration, the number of laccase genes in *S. bicolor* genome was finally predicted to be 27, i.e., *SbLAC1* to *SbLAC27* numbered according to their distribution on chromosomes 1, 2, 3, 4, 5, 8, 9, 10. Generally, SbLACs consist of 500–600 amino acids and the majority were probably secreted proteins as indicated by a cleavable N-terminal signal peptide, with a few exceptions predicted to be located in chloroplast (SbLAC3) or mitochondria (SbLAC9, SbLAC14, SbLAC16, SbLAC17, and SbLAC25). Additionally, variable N- or O-glycosylation sites and phosphorylation sites were predicted to present in all SbLAC proteins, indicating potential post-translational modifications (Table 1).

**Table 1:** **A summary of ***S. bicolor*** laccases**.

**Gene**	**Locus name**	**Alias**	**Peptide length**	**Signal peptide length**	**Cleavage site**	**Predicted subcellular location**	**Potential glycosylation sites**		**Potential phosphorylation sites**		
							**N-Glyc**	**O-Glyc**	**Ser^P^**	**Thr^P^**	**Tyr^P^**
*SbLAC1*	Sobic.001G403100	Sb01g038130	600	32	ALA-EE	Secretory	6	16	6	9	1
*SbLAC2*	Sobic.001G422300	Sb01g039690	576	25	SHG-AT	Secretory	14	12	4	7	2
*SbLAC3*	Sobic.002G001300	Sb02g000300	530	–	**–**	Chloroplast	5	11	9	5	0
*SbLAC4*	Sobic.003G111900	Sb03g009410	598	28	SQA-AV	Secretory	6	9	7	11	5
*SbLAC5*	Sobic.003G231400	Sb03g028920	491	28	AQA-DV	Secretory	6	20	11	6	1
*SbLAC6*	Sobic.003G341500	Sb03g038550	568	25	ADA-EV	Secretory	8	13	3	7	3
*SbLAC7*	Sobic.003G352700	Sb03g039520	579	22	ASA-VE	Secretory	13	13	3	9	7
*SbLAC8*	Sobic.003G352800	Sb03g039530	579	27	TEG-AI	Secretory	10	11	3	8	7
*SbLAC9*	Sobic.003G353200	Sb03g039570	579	37	AAG-DT	Mitochondrion	11	13	4	9	10
*SbLAC10*	Sobic.003G357500	Sb03g039960	565	21	AQA-AT	Secretory	8	7	12	13	4
*SbLAC11*	Sobic.003G357600	Sb03g039970	649	22	ADA-AT	Secretory	5	22	16	7	2
*SbLAC12*	Sobic.003G357700	Sb03g039980	557	24	ANA-AV	Secretory	2	9	9	6	6
*SbLAC13*	Sobic.004G235900	Sb04g027850	591	29	AQA-SR	Secretory	4	7	12	7	8
*SbLAC14*	Sobic.004G236000	Sb04g027860	590	32	VLA-FG	Mitochondrion	5	8	9	9	5
*SbLAC15*	Sobic.004G236100	–	573	18	VLA-FG	Secretory	6	7	9	8	7
*SbLAC16*	Sobic.004G314200	Sb04g034610	603	40	AQA-SR	Mitochondrion	9	11	10	13	7
*SbLAC17*	Sobic.004G314300	Sb04g034620	591	–	**–**	Mitochondrion	9	12	9	9	5
*SbLAC18*	Sobic.005G005800	Sb05g000680	587	38	TIA-KE	Secretory	9	18	8	10	4
*SbLAC19*	Sobic.005G156700	Sb05g021890	554	23	VLA-FG	Secretory	6	12	8	9	7
*SbLAC20*	Sobic.005G163800	Sb05g022480	574	26	AQA-SR	Secretory	3	11	7	8	11
*SbLAC21*	Sobic.005G198500	Sb05g025570	601	21	STA-VS	Secretory	10	11	2	9	6
*SbLAC22*	Sobic.005G215300	Sb05g026630	600	21	SAA-TT	Secretory	7	13	8	11	3
*SbLAC23*	Sobic.008G006900	Sb08g000720	323	–	**–**	–	8	12	6	1	1
*SbLAC24*	Sobic.008G090800	Sb08g011530	576	25	AQA-SI	Secretory	10	16	8	17	5
*SbLAC25*	Sobic.009G162300	Sb09g022460	585	31	AAA-RT	Mitochondrion	13	15	4	11	4
*SbLAC26*	Sobic.009G162800	Sb09g022510	585	31	AEA-ET	Secretory	12	14	10	8	6
*SbLAC27*	Sobic.010G268500	Sb10g030340	605	36	VQA-ST	Secretory	4	8	10	12	1

### *S. bicolor* laccases had conserved copper-binding domains but potentially distinct functions

**Figure 1:**
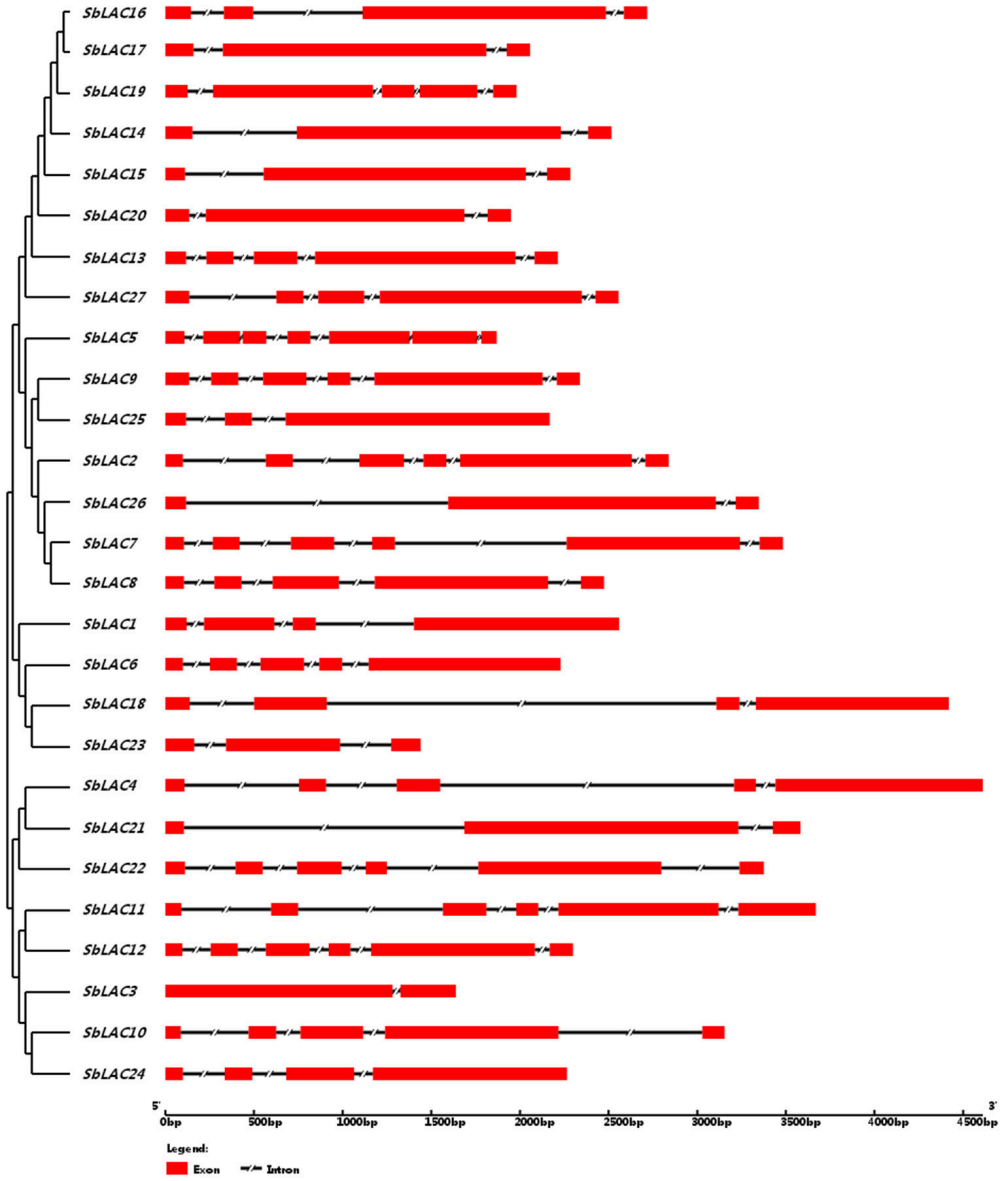
**The intron-exon patterns of ***S. bicolor*** laccase genes**. Exons and introns are represented by red rectangle and shrinked lines, respectively.

The structure of *S. bicolor* laccase genes exhibited diverse intron-exon patterns, as the number of exons ranged from 2 to 7 (Figure 1). Likewise, amino acid sequence alignment showed relatively low sequence identity, as the identity percentage varied from 28 to 80% for most ones, with the highest homology between SbLAC14 and SbLAC15 (86.51%). Though, the equivalent L1-L4 signature sequences that distinguish laccase within the broader class of multicopper oxidases were present among individual *S. bicolor* laccase members (Table 2). The amino acids potentially involved in copper binding, including ten histidines and one cysteine as well as an axial ligand of methionine or leucine, were housed in the four conserved regions (Table 2). A total of 11 *S. bicolor* laccases (SbLAC4, SbLAC13, SbLAC14, SbLAC15, SbLAC16, SbLAC17 SbLAC19, SbLAC20, SbLAC21, SbLAC22, and SbLAC27) were provisionally grouped into the low-redox potential class (Met as the axial ligand), while the others belong to the high-redox potential class (Leu as the axial ligand).

**Table 2:** **Conserved copper ligands in L1-L4 signature sequence**.

**Laccase**	**L1**	**L2**	**L3**	**L4**
	** 2 3**	** 3 3**	** 1 2 3**	** 313 1 1**
SbLAC1	90 HWHGLRQLRNGWADGPEFVTQCPI 113	130 GTLWWHAHSSWLRATVHGALIIHPRRG 156	499 HPMHIHGY 506	561 HCHIDAHLTGGL 572
SbLAC2	83 HWHGVRQLRTGWSDGPAYVTQCPI 106	123 GTLFWHAHVSWLRATLYGPIVILPKRG 149	477 HPLHLHGF 484	539 HCHLEVHTSWGL 550
SbLAC3	–	–	437 HPVHLHGF 444	493 HCHLDAHLPFGL 504
SbLAC4	86 HWHGVKQRLTCWADGAGMVTQCPI 109	126 GTLWWHSHVSILRATLHGIIIIRPKSG 152	494 NPMHLHGH 501	556 HCHFEFHIAMGM 567
SbLAC5	89 HCHGLKQRRNGWADGP———————— 104	107 GTLWWHAHIAWLRATVHGAVVVLPERG 133	397 HPFHLHGY 404	459HCHLE—————GL465
SbLAC6	83 HWHGIRQMRTGWADGPEFVTQCPI 106	123 GTLWWHAHSSWLRATVYGGLIIRPREN 149	469 HPIHIHGY 476	531 HCHLDVHITWGL 542
SbLAC7	85 HWHGIRQLRTGWADGPAYITQCPI 108	125 GTLWWHAHISWLRATVYGPLVVLPKLG 151	480 HPLHLHGF 487	542 HCHLEVHTTWGL 553
SbLAC8	85 HWHGVRQLRSGWADGPAYITQCPI 108	125 GTLWWHAHISWLRATVYGAIVILPKPG 151	480 HPLHLHGF 487	542 HCHLEVHVSWGL 553
SbLAC9	95 HWHGVRQLRNGWADGPAYITQCPI 118	135 GTLWWHAHFSWLRVHLYGPLVILPKRG 161	480 HPLHLHGY 487	542 HCHFDVHLSWGL 553
SbLAC10	80 HWHGVFQRGTPWADGPSMVTQCPI 103	120 GTLWWHAHSSFLRATVYGALIIRPRSG 146	466 HPMHLHGF 473	530 HCHIDAHLTIGL 541
SbLAC11	80 HWHGVLQLMTPWADGPSMVTQCPI 103	120 GTLWWHAHSSFLRATVYGAFIIRPRRG 146	450 HPIHLHGF 457	510 HCHLDPHVPMGL 521
SbLAC12	82 HWHGIFQLRSGWADGANMITQCPI 105	122 GTLWWHAHASMLRATIYGALIIKPRNG 148	459 HPIHLHGF 466	520 HCHFDMHLPLGL 531
SbLAC13	89 HWHGVDQPRNPWSDGPEFITQCPI 112	129 GTLWWHAHSDFDRNTVHGAIVIRPRRG 155	492 HPIHLHGF 499	554 HCHFERHMAWGM 565
SbLAC14	101 HWHGVDQPRNPWSDGPEYITQCPI 124	141 GTLWWHAHSDFDRATVHGAIVIHPKRG 167	491 HPMHLHGF 498	553 HCHFDRHTAWGM 564
SbLAC15	87 HWHGVDQPRNPWSDGPEYITQCPI 110	127 GTLWWHAHSEFDRATVHGAIVIHPKRG 153	474 HPMHLHGF 481	536 HCHFDRHTVWGM 547
SbLAC16	111 HWHDVDQPRNPWSDGPEYITQCPI 134	151 GTLWWHAHSDFDRATVHGAVVIHPKHG 177	504 HPMHLHGF 511	566 HCHFDRHTVWGM 577
SbLAC17	103 HWHGVDQPRNPWFDGPEYITQCPI 126	143 GTLWWHAHSDFDRATVHGAIVIS———— 165	492 HPMHLHGF 499	554 HCHFNRHMMWGM 565
SbLAC18	96 HWHGVRQMRTGWSDGPEFVTQCPI 119	136 GTLWWHAHSSWLRATVHGALLIRPRAG 162	488 HPIHLHGY 495	550 HCHLDVHITWGL 561
SbLAC19	93 HWHGVDQPRNPWSDGPEYITQCPI 116	133 GTLWWHAHSDFDRATVHGAIVVHPKRG 159	455 HPMHLHGF 462	517 HCHFDHHTVWGM 528
SbLAC20	95 HWHGVDQPRNPWSDGPEHITQCPI 118	135 GTLWWHAHSDYGRTTVHGVIVIRPKDD 161	475 HPMHLHGY 482	537 HCHIDIHMVWGM 548
SbLAC21	85 HWHGVRQLRSCWSDGAGFVTECPI 108	125 GTLWWHAHVTCLRATINGAFVIRPKDG 151	494 NPMHLHGY 501	556 HCHFEFHIVMGM 567
SbLAC22	87 HWHGVYQMRNCWNDGVPMVTQRPI 110	127 GTLWWHAHDAFLRGTIYGALIIRPRQG 153	499 NPMHLHGH 506	561 HCHFEFHLAMGM 572
SbLAC23	–	–	254 HPIHLHGY 261	286 HCHLDVHITWGL 297
SbLAC24	84 HWHGIFQRGTPWADGPTMVTQCPV 107	124 GTLWWHAHISYLRATVYGALVLRPRGG 150	477 HPMHLHGY 484	539 HCHFDAHLDLGL 550
SbLAC25	89 HWHGVRQLRSGWSDGPSFITQCPI 112	129 GTLWWHAHFSWLRATLYGPLVILPPRG 155	486 HPLHLHGY 493	548 HCHLDVHLSWGL 559
SbLAC26	89 HWHGVRQLLSGWADGPSYITQCPI 112	129 GTLWWHAHISWLRATVYGPIVILPPAG 155	486 HPLHLHGF 493	548 HCHLEVHMSWGL 559
SbLAC27	95 HWHGVDQPRNPWSDGPEYITQCPI 118	135 GTLWWHAHTGFDRATVHGAIVVLPRRG 161	506 HPMHLHGF 513	568 HCHFDRHMVWGM 579

*The equivalent signature sequences that distinguish S. bicolor laccases include L1, HWHGX_9_DGX_5_QCPI; L2, GTLWWHAHX_9_GX_5_PX_2_G; L3, HPXHLHGX; L4, HLHX_3_HX_3_GX. The amino acids potentially involved in copper binding were highlighted in red, with numbers 1, 2, and 3 corresponding to Cu1, Cu2, and Cu3 ions*.

**Figure 2:**
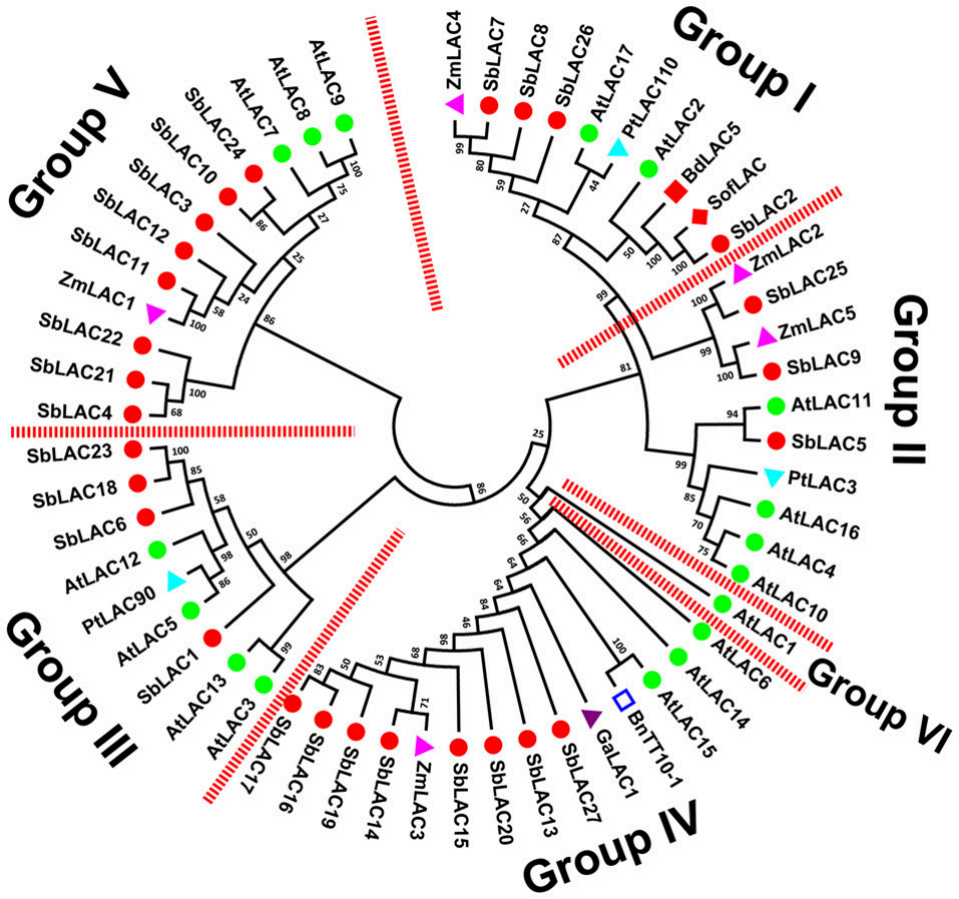
**Phylogeny of ***S. bicolor*** laccases with the ones previously characterized from other plants**. A total of fifty six plant laccases were used for MEGA analysis. 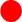, SbLAC1 to 27; 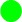, AtLAC1 to 17; 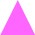, ZmLAC1 to 5; 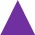, GaLAC1; 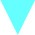, PtLAC3, PtLAC90, and PtLAC110; 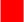, BdLAC5; 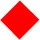, SofLAC; 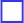, BnTT10-1.

*S. bicolor* laccases were clustered into six phylogenetic groups (Figure 2). To be concrete, SbLAC2, SbLAC7, SbLAC8, and SbLAC26 were clustered in Group I with AtLAC17, SofLAC and BdLAC5, while SbLAC5, SbLAC9, and SbLAC25 were gathered in Group II together with AtLAC4, AtLAC11, and PtLAC3, all the latter ones have been genetically proved to be monolignol laccases involved in lignin biosynthesis, implying similar role for SbLAC2, SbLAC7, SbLAC8, and SbLAC26 in Group I and SbLAC5 in Group II. Particularly, SbLAC2 was the closest homolog to SofLAC (95.59% identity) and BdLAC5 (84.95% identity), while SbLAC5 was also tightly clustered with AtLAC11 (a lower identity of 65.20%), thus added the probability of catalyzing lignin biosynthesis. Taken that monolignol laccases AtLAC4, AtLAC17, and BdLAC5 have been proved to be localized in secondary cell wall (Schuetz et al., 2014; Wang Y. et al., 2015), SbLAC9 and SbLAC25 were more likely to participate in mitochondria oxidation-reduction cycle or similar processes due to their predicted location in mitochondria. Interestingly, Group IV comprised eight sorghum laccases from the 11 putative low-redox potential members (except SbLAC4, SbLAC21, and SbLAC22), along with AtLAC15, BnTT10-1, GaLAC1, and ZmLAC3, all of which have similar low-redox potential and have been previously reported to take part in polymerization of phenolic compounds (Ranocha et al., 2002; Cai et al., 2006; Caparrós-Ruiz et al., 2006; Liang et al., 2006a; Zhang K. et al., 2013). The classification suggested that the eight members were likely to catalyze oxidation of phenolic compounds. Meanwhile, the remaining three with low-redox potential, as well as five other *S. bicolor* laccases, were distributed together in Group V with stress-induced AtLAC7, AtLAC8, AtLAC9, and ZmLAC1. Besides the above, Group III contained four SbLACs and three Arabidopsis laccase members with unknown functions. Group VI included only AtLAC1, with none of the 27 *S. bicolor* laccase members.

### *SbLAC* genes were preferentially expressed in roots and young internodes

**Figure 3:**
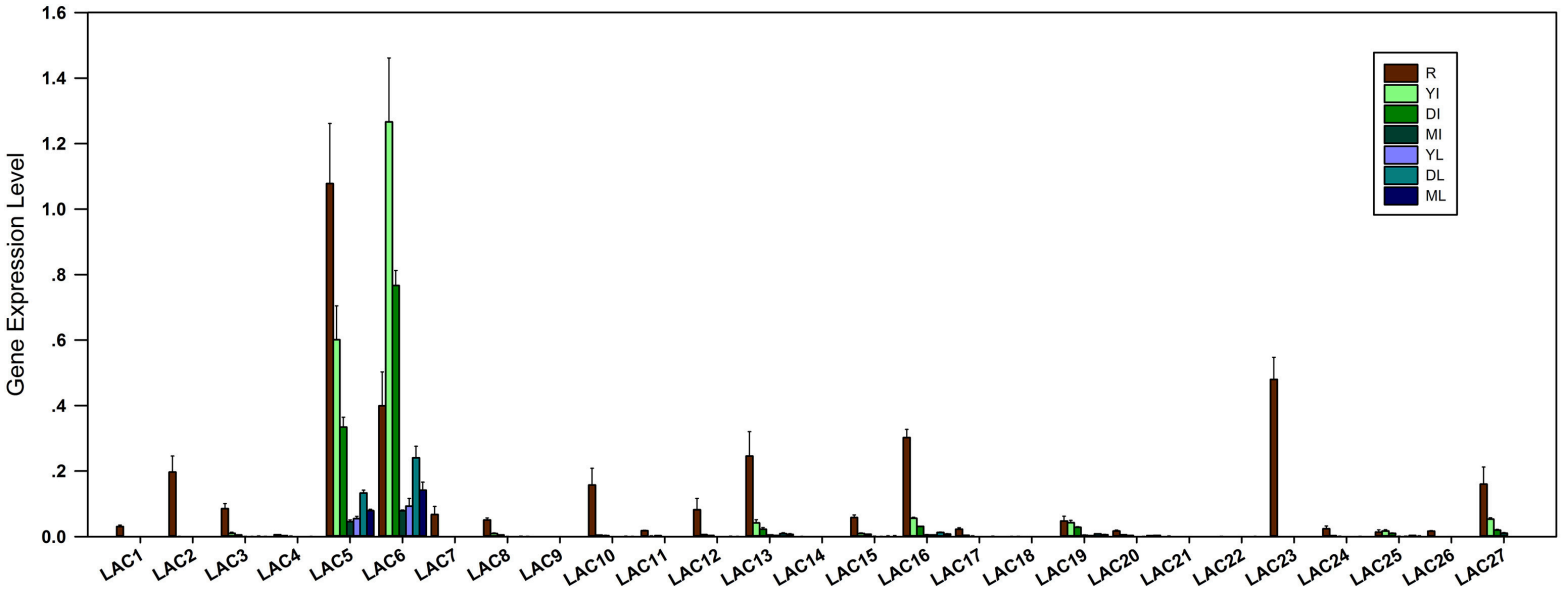
**Expression profiles of ***SbLAC***s in H18 internodes**. The expression levels are represented by means ± SD of three independent replicates. R, roots; YI, young internodes; DI, developing internodes; MI, mature internodes; YL, DL, and ML, leaves to corresponding young, developing and mature internodes, respectively.

To investigate the organ and development-specific expression patterns of *SbLAC* genes, qRT-PCR was used to detect the expression of *SbLACs* in roots, mature, developing and young internodes as well as the leaves to corresponding internodes. The results showed that *SbLAC* members have different expression levels and differential organ expression patterns. Among them, the expressions of *SbLAC4/9/14/18/21/22* were hardly detected. All the remaining members showed highest expression levels in roots except for *SbLAC6* and *SbLAC25*, which exhibited highest level in young internodes (Figure 3). Such pattern of highest expression of *LAC* genes in roots has been reported in *Arabidopsis* and *Z. mays* (McCaig et al., 2005; Caparrós-Ruiz et al., 2006; Abdel-Ghany and Pilon, 2008), which may be due to the main accumulation of copper ions in plant roots (Burkhead et al., 2009). Moreover, 13 *SbLACs* showed higher expression in young internodes but reduced level in pace with maturity (Figure 3), similarly to the previously reported sugarcane *SofLAC*, which was preferentially expressed in sclerenchymatic and parenchymatic cells of young internodes (Cesarino et al., 2013). This kind of expression pattern reconfirmed the hypothesis that laccases may function in early stages of lignification to polymerize monolignols into oligo-lignols (Sterjiades et al., 1993). Among all the *SbLAC* genes, *SbLAC5* and SbLAC6 were highly expressed (the expression of SbLAC6 was higher than that of SbLAC5), while the other ones exhibited lower abundance.

### Diverse *cis* regulatory elements were recognized in *SbLAC* promoters

Various numbers of putative *cis*-acting elements, including the core TATA box and CAAT box, were detected in the promoters of *S. bicolor* laccase genes by PlantCARE (Table 3). All 27 *SbLAC* promoter sequences had many light responsive elements, such as G-box (Arguello-Astorga and Herrera-Estrella, 1998), revealing an essential role of SbLACs in plant morphogenesis. Besides, there are three types of representative DNA regulatory elements: hormone responsive elements involved in response to various plant hormones, such as abscisic acid (ABA), auxin, ethylene, gibberellins (GA), methyl jasmonate (MeJA), salicylic acid; stress responsive elements responding to diverse biotic (fungal elicitor and herbivore) and abiotic (anaerobic induction, cold and dehydration, defense and stress, drought, heat stress, low temperature, and wound) stresses; tissue specific expressed elements related to endosperm-, meristem- or seed-specific activation, and regulation. Moreover, two classes of AC elements (AC-I and AC-II), which has been reported in *AtLAC* promoters (Turlapati et al., 2011), were also discovered in promoters of seven *SbLAC* genes.

**Table 3:** **Putative regulatory motifs in ***SbLACs*** promoters**.

**Laccase**	**Light responsive**	**Hormone responsive**						**Stress responsive**									**Tissue specific expression**		
		**Abscisic acid**	**Auxin**	**Ethylene**	**Gibberellin**	**MeJA**	**Salicylic acid**	**Anaerobic induction**	**Cold and dehydration**	**Defense and stress**	**Drought**	**Fungal elicitor**	**Heat stress**	**Herbivore**	**Low temperature**	**Wound**	**meristem**	**endosperm**	**seed**
*SbLAC1*	+		+		+	+	+	+		+	+	+				+	+	+	
*SbLAC2*	+	+	+			+	+			+	+	+		+		+		+	
*SbLAC3*	+	+			+	+		+		+	+						+	+	+
*SbLAC4*	+	+			+	+	+	+			+	+			+	+		+	+
*SbLAC5*	+	+			+	+				+	+	+	+					+	
*SbLAC6*	+	+			+	+	+	+			+	+					+	+	+
*SbLAC7*	+				+		+	+		+	+	+			+				+
*SbLAC8*	+			+	+			+			+	+	+		+		+		+
*SbLAC9*	+	+					+	+		+	+	+			+		+	+	
*SbLAC10*	+		+		+	+		+			+	+	+		+		+	+	
*SbLAC11*	+	+			+	+		+		+	+	+		+	+			+	
*SbLAC12*	+		+		+	+	+	+			+				+			+	
*SbLAC13*	+	+				+		+			+	+	+				+	+	
*SbLAC14*	+	+	+				+	+				+	+						+
*SbLAC15*	+	+	+		+	+	+	+		+	+							+	+
*SbLAC16*	+	+	+			+	+	+		+	+							+	+
*SbLAC17*	+	+			+	+		+	+	+		+						+	+
*SbLAC18*	+	+			+		+	+		+	+	+						+	+
*SbLAC19*	+	+	+	+	+		+	+	+		+	+		+	+		+	+	
*SbLAC20*	+			+	+	+	+	+			+	+	+			+		+	+
*SbLAC21*	+	+	+		+	+	+	+		+	+	+	+				+	+	+
*SbLAC22*	+	+			+	+	+			+	+	+			+			+	
*SbLAC23*	+	+	+		+	+		+		+	+	+	+		+		+	+	
*SbLAC24*	+		+		+	+		+		+	+	+	+				+	+	
*SbLAC25*	+	+				+	+				+	+	+				+	+	
*SbLAC26*	+	+	+		+			+			+							+	
*SbLAC27*	+				+	+		+		+	+	+	+		+	+	+	+	

### Seven *SbLACs* were found to be potential targets of sbi-miRNAs

Seven *SbLACs* were predicted to be potential sbi-miRNA targets (Details in Supplemental Table 4), among which *SbLAC8* was possibly targeted by sbi-miRNA164 members (a, b, d, and e) in its N-terminal signal peptide. The mode of action was nearly the same for sbi-miR528 targeted *SbLAC9* and *SbLAC21*. Additionally, *SbLAC2, SbLAC7, SbLAC8*, and *SbLAC26* were putative targets of sbi-miRNA397-5p and shared similar interaction with sbi-miR397-5p at the position of Cu-oxidase domain. In addition, sbi-miRNA6235-5p had only one target *SbLAC17*.

## Discussion

The availability of the whole genome sequences for grain sorghum facilitated characterization of laccase genes in sweet sorghum, since grain sorghum and sweet sorghum are clustered together in diversity studies despite certain genetic variation (Ritter et al., 2007; Zheng et al., 2011). Laccase is encoded by a multigene family in plants. For instance, the Arabidopsis genome encodes 17 laccases dispersed across chromosome 1, 2, 3, and 5, and the number of putative laccase genes in *S. officinarum* and *B. distachyon* is 12 and 29, respectively (McCaig et al., 2005; Cesarino et al., 2013; Wang Y. et al., 2015). Here, we characterized 27 laccase candidates in *S. bicolor*. They exhibited the typical characteristics of three conserved Cu-oxidase domains, four signature sequences and twelve housed copper ligands. Even so, relatively low sequence similarities and quite different intron-exon structures were presented among the 27 members, implicating potentially functional divergence.

### *S. bicolor* internodes were favorable for identification of cell-wall related genes

Internodes of grass stalks have been considered as a useful model for identification of cell wall-related genes, since the successive internodes from the apex to the base represent a developmental profile of young to mature (Sattler et al., 2009; Bosch et al., 2011). Therefore, we chose internodes 2, 6, and 12 from the bottom up in sweet sorghum plants as representative samples belonging to different developmental stages. Case studies of histochemical staining of sugarcane and maize internodes indicated that lignification in young internodes was restricted to tracheary elements while in developing and mature internodes, lignin accumulated significantly in different cell types (Bosch et al., 2011; Cesarino et al., 2012), establishing the association of internodes anatomical changes with lignification during plant development. On the other hand, it has been reported that the expression of genes involved in lignin biosynthetic pathway, including *PAL, 4CL, CCR, CCoAOMT, F3'H, LAC, etc*., were higher in mature internodes with active secondary cell wall synthesis and lignifications (Sattler et al., 2009), which build the basis for functional analysis of lignin-related genes within mature internodes in plants with stalks that are attached to grass family.

### *SbLAC5* and *SbLAC6* were *bona fide* correlated to lignin biosynthesis

It would be a formidable task to determine the exact role of each SbLAC member on account of expression and functional redundancy, but hints can be acquired from phylogenetic analysis. For example, eight SbLAC members were clustered with stress-induced AtLAC7, AtLAC8, AtLAC9, and ZmLAC1. *AtLAC7* was reported to be up-regulated under iron deficiency while *AtLAC8* and *AtLAC9* were induced by 150 mM NaCl treatment and wounding in Arabidopsis (McCaig et al., 2005). And in maize primary roots treated with varied NaCl concentrations, increase of *ZmLAC1* transcripts was observed (Liang et al., 2006b). The clustering revealed potential involvement of sorghum laccases in responses to environmental stresses. In another research of our laboratory (Feng et al., unpublished data), changed or different expression of *SbLAC4, SbLAC21*, and *SbLAC22* genes has been detected in 10 μM Cd treated sweet sorghum H18 and L69, indicating that the three laccases may participates in Cd stress response.

The role of catalyzing lignin biosynthesis has always been expected to be acted by class III peroxidases, while the demonstration of laccases functioning in monolignols polymerization has raised the viewpoint that laccases might act redundantly with peroxidases. However, simultaneous disruption of the Arabidopsis *LAC4, LAC11*, and *LAC17* brought almost abolished lignin deposition and severe growth defect in *lac4lac11lac17* triple mutant, while casparian strip was still lignified through the activity of peroxidase, suggesting that laccase is necessary and non-redundant with peroxidase for lignin polymerization during vascular development in Arabidopsis (Zhao et al., 2013). It has been suggested by Sterjiades that laccase might function during early lignification stages whereas cell-wall peroxidases play the role in the follow-up proceedings of xylem development (Sterjiades et al., 1993), which can partially explain the reduced expression of *SbLACs* following maturity of internodes.

Based on phylogenetic analysis, SbLAC2 and SbLAC5 were considered to catalyze lignin biosynthesis. The results of qRT-PCR detected high abundance of *SbLAC5* transcripts, making it the closest lignin-specific candidate (Figure 3). What's more, it's interesting to find that *SbLAC6* also showed high expression, indicating a necessary but unclear role in lignin formation. In order for functional verification, more researches such as *in situ* hybridization or fluorescence microscopy should be performed for accurate protein localization. Examples of references are limited but come to a similar conclusion that monolignol laccases are potentially localized in apoplast of lignified tissues. It has been reported that AtLAC4 and AtLAC17 are located in secondary cell walls throughout protoxylem tracheary element differentiation in Arabidopsis (Schuetz et al., 2014). And in *B. distachyon*, BdLAC5 were detected in apoplasm in lignified interfascicular fibers (Wang Y. et al., 2015). Besides, genetic evidence is indispensable from either heterologous expression or self-transformation. The demonstration of *bona fide* SbLACs catalyzing lignin biosynthesis will point out the direction of generating feedstock with genetically alleviated recalcitrance but improved digestibility and bioethanol yields.

### Regulation of *SbLACs*

While much progress has been made in characterization of plant laccase genes, less is clearly defined concerning the precise regulatory mechanisms. We proposed that the expression of SbLACs may be regulated by transcription factors at transcriptional level, by sbi-miRNAs at post-transcriptional level or by post-translational modifications.

Putative interaction of SbLACs with transcription factors (TFs) can be indicated by varied *cis* elements in *SbLAC* promoter sequences. For example, the G-box elements are usually present in promoters of light-responsive genes, serving as binding sites for bZIP, bHLH, and NAC TFs (Toledo-Ortiz et al., 2003; Guo and Gan, 2006; Shen et al., 2008). They have been reported to confer salt tolerance in plants, and play roles in Arabidopsis jasmonate (JA) response and early senescence of rice flag leaf and so on. Besides, it has been reported in Arabidopsis that MYB58 was able to bind to AC elements and directly activate the expression of *LAC4* gene (Zhou et al., 2009). The existence of diverse cis elements in promoters of *SbLAC* genes provides valuable tips in future understanding of mechanisms regulating *SbLAC* expression.

It has been reported as a common mechanism in flowering plants that miRNA can negatively regulate laccase expression by degrading target mRNA. In Arabidopsis, seven laccases were validated to be targets for miR408, miR397, and miR857 under Cu deficient conditions (Abdel-Ghany and Pilon, 2008). Similarly in *P. trichocarpa* and *O. Sativa*, Ptr-miR397a and Os-miR397 were verified as negative regulators of *PtrLACs* and *OsLAC*, respectively (Lu et al., 2013; Zhang Y. C. et al., 2013). These works together suggest the strategy of indirect modification of *laccase* expression through modulating miRNAs expression. Here in our work, seven SbLACs were predicted to be sbi-miRNA targets, among which *SbLAC2, SbLAC7, SbLAC8*, and *SbLAC26* were clusterd within Group I in phylogenetic tree, together with lignin-related *AtLAC17, BdLAC5*, and *SofLAC*, suggesting the role of sbi-miRNA397-5p in modulating cell wall lignin biosynthesis. Therefore, it can be applied to engineer sorghum lignin profile via genetic manipulation of sbi-miRNA397-5p by reference to Lu et al. and Wang et al., where overexpression of Ptr-miR397a and miR397b led to reduced lignin content in transgenic *P. trichocarpa* and Arabidopsis, respectively (Lu et al., 2013; Wang et al., 2014). What is important to note is that the putative sbi-miRNA/laccase pairs should be experimentally confirmed by modified 5′-rapid amplification of cDNA ends (RACE). Genetic evidences are essentially required as well.

Plant laccases are glycoproteins with relatively high carbohydrate content (20–45%) (Wang J. H. et al., 2015). It's not a surprise to find variable N- or O-glycosylation sites in *S. bicolor* laccase proteins, which might be effective in copper retention, enzyme stability (Ceriotti et al., 1998) and activity (Graziani et al., 1990). In addition, there exist a number of predicted serine, threonine, and tyrosine phosphorylation sites, symbols of underlying regulation by phosphorylation (Johnson and Lewis, 2001). Protein phosphorylation is one of the most widespread post-translational modifications that regulate protein activity, location, stability, or interactions (Guérinier et al., 2013; Meng et al., 2013; Umezawa et al., 2013). Numerous phosphorylation events have been identified in cellulose biosynthetic CESA proteins and CSC-associated subunits (Taylor, 2007; Chen et al., 2010; Bischoff et al., 2011; Jones et al., 2016), whereas the involvement of phosphorylation in monolignol biosynthesis has been rarely demonstrated. It was discovered only in *P. trichocarpa* that phosphorylation performed as an on/off switch for 5-hydroxyconiferaldehyde O-methyltransferase (PtrAldOMT2) activity in poplar monolignol biosynthesis (Wang J. P. et al., 2015). Elucidation of phosphorylation patterns in laccases will add new clues to the regulatory network for plant lignin biosynthetic pathway.

To sum up, plant laccase has drawn more and more attention in recent years with focus on its involvement in lignin biosynthesis, making it an ideal target for lignocellulose modification, especially in those bioenergy plants. It should be valuable to engineer sweet sorghum biomass through manipulation of laccase or sbi-miRNA for fine-tuning lignin profile. Our work adds to the knowledge for genetic engineering of sweet sorghum, and demonstrates a promising future in sweet sorghum cultivation for biofuel production on marginal lands such as saline or heavy metal polluted soils.

## Author contributions

YL and SL initiated the research. JF, JW, and YL designed the experiments. JW performed the experiments, analyzed the data, and drafted the manuscript. JF and YL helped draft and revise the manuscript. WJ contributed to plant material cultivation. PF and HB participated in revising the manuscript. All authors read and approved the final manuscript.

## Funding

This work was financially supported by the Key Project of Intergovernmental International Cooperation on S&T Innovation, National Key R&D Program (Project number: S2016G9072).

### Conflict of interest statement

The authors declare that the research was conducted in the absence of any commercial or financial relationships that could be construed as a potential conflict of interest.

## Abbreviations

4CL, 4-coumarate: CoA: ligase
miRNA: micro-RNA
C4H: cinnamate 4-hydroxylase
CAD: cinnamyl alcohol dehydrogenase
CCR: (hydroxy)cinnamoyl CoA reductase
COMT: caffeic acid (5-hydroxyconiferaldehyde) O-methyltransferase
FA: ferulic acid
G: guaiacyl
H: *p*-hydroxyphenyl
H18: high-Cd accumulation genetype UMM EL TEIMAN
HCT: hydroxycinnamoyl CoA shikimate hydroxycinnamoyl transferase
Leu: leucine
Met: methionine
MCOs: multicopper oxidases
NCBI: National Center for Biotechnology Information
PAL: phenylalanine ammonia lyase
PlantCARE: Plant *cis*-Acting Regulatory Elements
RACE: rapid amplification of cDNA ends
S: syringyl
SMART: Simple Modular Architecture Research Tool
TF: transcription factors.
