# Supplementary material for: Genome-Wide Identification of Sorghum bicolor Laccases Reveals Potential Targets for Lignin Modification
Source: Front Plant Sci. 2017 May 5;8:714. doi: 10.3389/fpls.2017.00714 (PMC5418363; doi:10.3389/fpls.2017.00714)
Supplement: Supplementary file 1 [file Table1.DOCX]

**Supplemental Table 1 Database accession numbers of plant laccases**

| **Plant** | **Laccase** | **Accession number** |
| --- | --- | --- |
| ***Arabidopsis thaliana*** | AtLAC1 | [TAIR: AT1G18140] |
|  | AtLAC2 | [TAIR: AT2G29130] |
|  | AtLAC3 | [TAIR: AT2G30210] |
|  | AtLAC4 | [TAIR: AT2G38080] |
|  | AtLAC5 | [TAIR: AT2G40370] |
|  | AtLAC6 | [TAIR: AT2G46570] |
|  | AtLAC7 | [TAIR: AT3G09220] |
|  | AtLAC8 | [TAIR: AT5G01040] |
|  | AtLAC9 | [TAIR: AT5G01050] |
|  | AtLAC10 | [TAIR: AT5G01190] |
|  | AtLAC11 | [TAIR: AT5G03260] |
|  | AtLAC12 | [TAIR: AT5G05390] |
|  | AtLAC13 | [TAIR: AT5G07130] |
|  | AtLAC14 | [TAIR: AT5G09360] |
|  | AtLAC15 | [TAIR: AT5G48100] |
|  | AtLAC16 | [TAIR: AT5G58910] |
|  | AtLAC17 | [TAIR: AT5G60020] |
| ***Brachypodium distachyon*** | BdLAC5 | [Phytozome: Bradi1g66720] |
| ***Brassica napus*** | BnTT10-1 | [Genbank: AEK27149] |
| ***Gossypium arboreum*** | GaLAC1 | [Genbank: AAR83118] |
| ***Populus trichocarpa*** | PtLAC3 | [EMBL: [CAA74103](http://www.ebi.ac.uk/ebisearch/redirect.ebi?url=http%3A%2F%2Fwww.ebi.ac.uk%2Fena%2Fdata%2Fview%2FCAA74103&digest=BAB509)] |
|  | PtLAC90 | [EMBL: [CAA74104](http://www.ebi.ac.uk/ebisearch/redirect.ebi?url=http%3A%2F%2Fwww.ebi.ac.uk%2Fena%2Fdata%2Fview%2FCAA74103&digest=BAB509)] |
|  | PtLAC110 | [EMBL: [CAA74105](http://www.ebi.ac.uk/ebisearch/redirect.ebi?url=http%3A%2F%2Fwww.ebi.ac.uk%2Fena%2Fdata%2Fview%2FCAA74103&digest=BAB509)] |
| ***Saccharum officinarum*** | SofLAC | [SUCEST: SCUTST3084C11.g] |
| ***Zea mays*** | ZmLAC1 | [Genbank: AAX83112] |
|  | ZmLAC2 | [EMBL: CAJ30498] |
|  | ZmLAC3 | [EMBL: CAJ30499] |
|  | ZmLAC4 | [EMBL: CAJ30500] |
|  | ZmLAC5 | [EMBL: CAJ30497] |
