# Supplementary material for: Genome-Wide Identification of Sorghum bicolor Laccases Reveals Potential Targets for Lignin Modification
Source: Front Plant Sci. 2017 May 5;8:714. doi: 10.3389/fpls.2017.00714 (PMC5418363; doi:10.3389/fpls.2017.00714)
Supplement: Supplementary file 2 [file Table2.DOCX]

**Supplemental Table 2 List of primers for qRT-PCR analysis**

| **Primer** | **Sequence (5’ to 3’)** |
| --- | --- |
| *18S rRNA* | F: CGAAAGTTGGGGGCTCGAAG |
|  | R: CCCCGGAACCCAAAGACTTTG |
| *SbLAC1* | F: CACTACCCTCCCTCTCCATCGG |
|  | GGCTTGTTTCTCTGGTGGTTTGCT |
| *SbLAC2* | F: ACTACCTGTCTACCTCGCCG |
|  | R: GCTCGTCGTGAACGATGTCA |
| *SbLAC3* | F: CAAAAAGCAGAAACACGCTGAGAAG |
|  | R: CAACAAGGCTGCGATCGAGATG |
| *SbLAC4* | F: TGGTGAATTAAAGCAAAACCTTG |
|  | R: TGGTTTTCAATTGGTAGGAGTAGTG |
| *SbLAC5* | F: GACCGGCACCTGCTGTACACCAT |
|  | R: CTTGCTCAGCCTCGTGCCCAG |
| *SbLAC6* | F: TTGCTCCTGCCCTCGAACGC |
|  | R: CGGCGTTGGCTCTCGATGG |
| *SbLAC7* | F: GCCCAAGCTGCGTTTCTGTC |
|  | R: CGAGGAGATGGAGGAATAATGTGTT |
| *SbLAC8* | F: GATACAGCCTTCCGCCAAGT |
|  | R: TTTTGTTCCACCACTCGCCT |
| *SbLAC9* | F: CAAACACACATAAGAAGCAGGGCG |
|  | R: GGAAGGGACATGCGGAGAAGAC |
| *SbLAC10* | F: CTTTTAGTTTTCCATGCACGTCTTG |
|  | R: GGAGAATTGGAGCTTTGCAAGG |
| *SbLAC11* | F: CCAAGTCATTCCTCCAGAACTCACC |
|  | R: GCGGAGTCAGAGAAGGGAGGAAG |
| *SbLAC12* | F: GATACAGCCTTCCGCCAAGT |
|  | R: TTTTGTTCCACCACTCGCCT |
| *SbLAC13* | F: AGCCTGAACAACGTGAGCTT |
|  | R: GGGTTGCTAGGGAACAGGTC |
| *SbLAC14* | F: TCCTCTCGCTCGCTGCTAAACAC |
|  | R: GAGCTCGATCAGCTCTCCTGATCTG |
| *SbLAC15* | F: GCCTTTGCGTTCTCGCTTGCT |
|  | R: GGGGCAAGCAGCAGAGCTAGAG |
| *SbLAC16* | F: TGCTGGTTAATGCTAGGCGT |
|  | R: CGGTTCACAGCCAGGATGAT |
| *SbLAC17* | F: GGTGGCGGCACGACGAACTAT |
|  | R: CAGCGTGCGAGCGATGGAG |
| *SbLAC18* | F: CCACAAGACCACCGCTGCCTAT |
|  | R: CAAGAGGCCAAGGCTCAAGACTCTG |
| *SbLAC19* | F: CGCACGACTACTCCCGTATGATGAT |
|  | R: CGGCTTGTTGGGGAAGTCCAG |
| *SbLAC20* | F: TTAAGCCGGTGTAGCTAAGATGCC |
|  | R: ACTGTTCAAGCAGCAACGAATTCC |
| *SbLAC21* | F: CTAGCGCCACTGTTGTTGCCAT |
|  | R: CGCCAATGGAAACTGCCGTG |
| *SbLAC22* | F: GCTTCAAACATTGTTGTCCCCCAT |
|  | R: GCGTTATGGTAGTAGCGGCGG |
| *SbLAC23* | F: TCATTCATTTCAACATCGACTGCGT |
|  | R: CCGGGGGCCTGTCATTAATTTAG |
| *SbLAC24* | F: ACCCAATCACGCGTACATATAAGAGT |
|  | R: CCAGCAATGACCAAAGACACAGG |
| *SbLAC25* | F: CCAACCACTATCGCTCTGTGACCTC |
|  | R: CGCCTTGCTGCTCGATCTGC |
| *SbLAC26* | F: GAGAGCCATCCAATGCACCT |
|  | R: AAAGTGGCAATGCACGAACC |
| *SbLAC27* | F: CCCCTCCTGCCTATAAATTGACTG |
|  | R: ACTCCAAAGGCCAAGAGCACAG |
