# Supplementary material for: Genome-Wide Identification of Sorghum bicolor Laccases Reveals Potential Targets for Lignin Modification
Source: Front Plant Sci. 2017 May 5;8:714. doi: 10.3389/fpls.2017.00714 (PMC5418363; doi:10.3389/fpls.2017.00714)
Supplement: Supplementary file 3 [file Table3.DOCX]

**Supplemental Table 3: The 57 transcripts derived from blastP search.** They were re-blasted in NCBI and were checked for Cu-oxidase domain on SMART, which indicated that 9 were monocopper oxidase-like proteins, 15 were *L*-ascorbate oxidase homologs, while only 33 were considered as laccase candidates.

**Supplemental Table 3 The 57 peptide sequences derived from BlastP search**

|  | **Laccase candidates** |
| --- | --- |
|  | Sobic.001G403100.1.p |
|  | Sobic.001G422300.1.p |
|  | Sobic.002G001300.3.p |
| **Monocopper oxidase-like proteins** | Sobic.003G111900.1.p |
| Sobic.001G369800.1.p | Sobic.003G111900.2.p |
| Sobic.001G199440.1.p | Sobic.003G231400.1.p |
| Sobic.003G088500.1.p | Sobic.003G341500.1.p |
| Sobic.003G088800.1.p | Sobic.003G352700.1.p |
| Sobic.007G043300.2.p | Sobic.003G352800.1.p |
| Sobic.007G043300.3.p | Sobic.003G353200.1.p |
| Sobic.007G043300.4.p | Sobic.003G357500.1.p |
| Sobic.007G043300.5.p | Sobic.003G357500.2.p |
| Sobic.010G003100.1.p | Sobic.003G357500.3.p |
| **L-ascorbate oxidase homologs** | Sobic.003G357500.4.p |
| Sobic.002G016400.2.p | Sobic.003G357500.5.p |
| Sobic.002G314800.1.p | Sobic.003G357600.1.p |
| Sobic.002G183600.1.p | Sobic.003G357700.1.p |
| Sobic.002G183700.1.p | Sobic.003G357700.2.p |
| Sobic.002G183800.1.p | Sobic.004G235900.1.p |
| Sobic.003G014600.1.p | Sobic.004G236000.1.p |
| Sobic.003G014766.1.p | Sobic.004G236100.1.p |
| Sobic.003G335200.1.p | Sobic.004G314200.1.p |
| Sobic.006G178400.1.p | Sobic.004G314300.1.p |
| Sobic.009G177300.1.p | Sobic.005G005800.1.p |
| Sobic.010G171800.1.p | Sobic.005G156700.1.p |
| Sobic.010G171800.2.p | Sobic.005G163800.1.p |
| Sobic.010G171900.1.p | Sobic.005G198500.1.p |
| Sobic.010G171900.2.p | Sobic.005G215300.1.p |
| Sobic.010G230000.1.p | Sobic.008G006900.1.p |
|  | Sobic.008G090800.1.p |
|  | Sobic.009G162300.1.p |
|  | Sobic.009G162800.1.p |
|  | Sobic.010G268500.1.p |
