# Supplementary material for: Genome-Wide Identification of Sorghum bicolor Laccases Reveals Potential Targets for Lignin Modification
Source: Front Plant Sci. 2017 May 5;8:714. doi: 10.3389/fpls.2017.00714 (PMC5418363; doi:10.3389/fpls.2017.00714)
Supplement: Supplementary file 4 [file Table4.DOCX]

**Supplemental Table 4: Sbi-miRNAs and the potential target laccase genes.** Sbi-miRNA sequences and the complementary laccase mRNA sequences are shown. Watson-Crick pairing (colons) or G-U wobble pairing (dots) is indicated.

**Supplemental Table 4 Sbi-miRNAs and the potential target laccase genes**

| **miRNA Acc.** | **Target Acc.** | [**Expectation (E)**](http://plantgrn.noble.org/psRNATarget/?dowhat=Help#maxexpectation) | [**Target Accessibility (UPE)**](http://plantgrn.noble.org/psRNATarget/?dowhat=Help#upe) | **Alignment** | [**Inhibition**](http://plantgrn.noble.org/psRNATarget/?dowhat=Help#validcleavageregion) |
| --- | --- | --- | --- | --- | --- |
| sbi-miR164a | *SbLAC8* | 3.0 | 20.78 | miRNA 21 ACGUGCACGGGACGAAGAGGU 1  ::: :::::.:: :::::::.  Target 373 UGCUCGUGCUCUUCUUCUCCG 393 | Translation |
| sbi-miR164b | *SbLAC8* | 3.0 | 20.78 | miRNA 20 CGUGCACGGGACGAAGAGGU 1  :: :::::.:: :::::::.  Target 374 GCUCGUGCUCUUCUUCUCCG 393 | Translation |
| sbi-miR164d | *SbLAC8* | 3.0 | 20.78 | miRNA 21 ACGUGCACGGGACGAAGAGGU 1  ::: :::::.:: :::::::.  Target 373 UGCUCGUGCUCUUCUUCUCCG 393 | Translation |
| sbi-miR164e | *SbLAC8* | 3.0 | 20.78 | miRNA 21 ACGUGCACGGGACGAAGAGGU 1  ::: :::::.:: :::::::.  Target 373 UGCUCGUGCUCUUCUUCUCCG 393 | Translation |
| sbi-miR397-5p | *SbLAC8* | 0.5 | 17.382 | miRNA 21 GUAGUUGCGACGUGAGUUACU 1  ::::::::::::.::::::::  Target 1010 CAUCAACGCUGCGCUCAAUGA 1030 | Cleavage |
| sbi-miR397-5p | *SbLAC26* | 2.5 | 19.859 | miRNA 21 GUAGUUGCGACGUGAGUUACU 1  ::::::::: :::::::: ::  Target 1198 CAUCAACGCCGCACUCAACGA 1218 | Cleavage |
| sbi-miR397-5p | *SbLAC2* | 3.0 | 19.126 | miRNA 21 GUAGUUGCGACGUGAGUUACU 1  ::::::::: ::.::::: ::  Target 804 CAUCAACGCCGCGCUCAACGA 824 | Cleavage |

| sbi-miR397-5p | *SbLAC7* | 3.0 | 23.636 | miRNA 21 GUAGUUGCGACGUGAGUUACU 1  ::::::::: ::.::::: ::  Target 1089 CAUCAACGCGGCGCUCAACGA 1109 | Cleavage |
| --- | --- | --- | --- | --- | --- |
| sbi-miR528 | *SbLAC9* | 3.0 | 15.109 | miRNA 21 GAGGAGACGUACGGGGAAGGU 1  ::.::: :::::.:::::::  Target 103 CUUCUCCGCAUGUCCCUUCCU 123 | Cleavage |
| sbi-miR528 | *SbLAC21* | 3.0 | 21.596 | miRNA 20 AGGAGACGUACGGGGAAGGU 1  :::::::..::.::::::.  Target 82 UCCUCUGUGUGUCCCUUCUU 101 | Cleavage |
| sbi-miR6235-5p | *SbLAC17* | 3.0 | 10.969 | miRNA 24 CGGUUGUCAUAAAAAGAGAGUGUU 1  ::::.:: :.::::::::::..::  Target 2256 GCCAGCAAUGUUUUUCUCUCGUAA 2279 | Cleavage |
